# Diffusion-Weighted Whole-Body Magnetic Resonance Imaging with Background Body Signal Suppression for Differentiating Infectious from Non-Infectious Aortitis

**DOI:** 10.3390/diagnostics16020225

**Published:** 2026-01-10

**Authors:** Jien Saito, Masahiro Muto, Masafumi Tada, Isao Yokota, Shinji Kamiya, Yukihide Numata, Hideki Sasaki, Takuya Hashizume, Kenji Iwata, Miki Asano, Satoru Wakasa

**Affiliations:** 1 Department of Cardiovascular Surgery, Hokkaido University Graduate School of Medicine, Sapporo 060-8648, Hokkaido, Japan; wakasa@med.hokudai.ac.jp; 2 Department of Cardiovascular Surgery, Nagoya City University East Medical Center, Nagoya 464-8547, Aichi, Japan; skamiya115@yahoo.co.jp (S.K.); yukihidenumata@msn.com (Y.N.); hideki.sasaki.jp@gmail.com (H.S.); asano@med.nagoya-cu.ac.jp (M.A.); 3 Department of Biostatistics, Hokkaido University Graduate School of Medicine, Sapporo 060-8638, Hokkaido, Japan; yokotai@pop.med.hokudai.ac.jp; 4 Department of Radiology, Ogaki Municipal Hospital, Ogaki 503-8502, Gifu, Japan; 5 Department of Health Promotion and Human Behavior, School of Public Health, Kyoto University Graduate School of Medicine, Kyoto 606-8501, Kyoto, Japan; tada.masafumi.76r@kyoto-u.jp; 6 Department of Neurology, Nagoya City University East Medical Center, Nagoya 464-8547, Aichi, Japan; 7 Department of Radiology, Nagoya City University East Medical Center, Nagoya 464-8547, Aichi, Japan; tky300@gmail.com (T.H.); k.iwata.medical@gmail.com (K.I.)

**Keywords:** whole-body imaging, aortitis, communicable diseases, diffusion magnetic resonance imaging, computed tomography

## Abstract

**Background/Objectives:** This study examined the clinical utility of diffusion-weighted whole-body magnetic resonance imaging with background body signal suppression (DWIBS) for differentiating infectious from non-infectious aortitis. **Methods:** The study included 32 patients with suspected inflammatory aortitis who underwent non-contrast computed tomography (NCCT) and magnetic resonance imaging. We evaluated the diagnostic performance of DWIBS using the spinal cord as a reference, NCCT, and their combination. The diagnosis of infectious aortitis was adjudicated based on imaging, clinical, and laboratory findings. We conducted a sensitivity analysis using a stricter definition of infectious aortitis that required both surgical and microbiological confirmation. **Results:** Fifteen patients were diagnosed with infectious aortitis. The sensitivity, specificity, and areas under the receiver operating characteristic curves were 93.3%, 70.6%, and 0.82, respectively, for NCCT; 93.3%, 76.5%, and 0.85, respectively, for DWIBS; and 86.7%, 94.1%, and 0.90, respectively, for the combination of both modalities. In the sensitivity analysis, the combined DWIBS and NCCT approach demonstrated a specificity of 87.5% and a sensitivity of 70.8%. **Conclusions:** DWIBS using the spinal cord as a reference appears to be a promising diagnostic tool for differentiating infectious from non-infectious aortitis, especially when combined with NCCT.

## 1. Introduction

Infectious aortitis, encompassing both native infectious aortic aneurysms (IAA) and aortic graft infection (AGI), is a life-threatening condition, increasing susceptibility to rupture and sepsis [1]. It has a high mortality rate of 26–44% [2]. Therefore, early and appropriate treatment should be performed based on rapid and reliable diagnosis. In particular, for aortic graft infections, precise diagnosis is crucial, as these conditions often require invasive treatment [3]. However, definitively diagnosing infectious aortitis using standard imaging modalities, including computed tomography (CT), remains challenging [4]. Therefore, diagnostic treatment is sometimes administered in cases of suspected conditions [5]. Positron emission tomography/CT (PET/CT) has emerged as a more precise diagnostic tool for aortic infections [6,7,8]. However, PET/CT use in Japan is restricted by limited availability, off-label status, and logistical challenges in acute settings. Therefore, developing a more feasible and accessible imaging modality for infectious aortitis is desired.

Diffusion-weighted whole-body imaging with background body signal suppression (DWIBS) has recently shown promise as an effective diagnostic tool for various conditions, including infectious aortitis. DWIBS was initially developed to evaluate primary tumors and metastases in cancer and has demonstrated diagnostic performance comparable to that of PET/CT [9,10,11,12]. Its utility in evaluating the activity of Takayasu arteritis, spine and soft tissue infections, and IAA has also been reported [13,14,15,16,17]. DWIBS is considered effective for identifying inflammation of the aorta; however, no reports have evaluated its diagnostic performance for infectious aortitis in a systematic manner. Therefore, this exploratory study assessed the diagnostic performance of DWIBS, alone and combined with NCCT, for infectious aortitis in a consecutive single-center cohort.

## 2. Materials and Methods

### 2.1. Study Design

**Figure 1:**
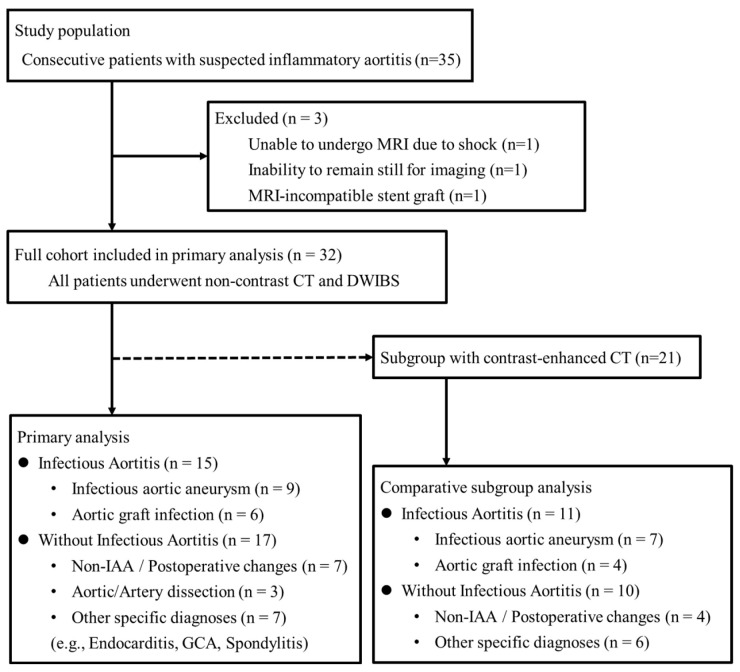
Flow diagram of patient enrollment. The diagram shows the selection of the study cohort from patients with suspected inflammatory aortitis, the reasons for exclusion, and the derivation of the subgroup for comparative analysis. The dashed line indicates the subset of patients included in the subgroup analysis. CT, computed tomography; MRI, magnetic resonance imaging; IAA, Infectious aortic aneurysm; GCA; giant cell arteritis.

This was a single-center retrospective study. Thirty-five consecutive patients with suspected IAA and/or AGI and/or inflammatory aortitis were referred to Nagoya City University East Medical Center between September 2020 and November 2022. Among them, 32 patients who underwent whole-body magnetic resonance imaging (MRI), including DWIBS, within 4 days before or after CT were included. Patients were excluded if they were unable to undergo an MRI or refused participation for any reason. Three patients were excluded because they were unable to undergo MRI due to shock, inability to remain still, or a previously implanted MRI-incompatible stent graft (Zenith; Cook Medical, Bloomington, IN, USA) (Figure 1). Based on the final diagnosis, the study cohort consisted of 32 patients, including 15 with infectious aortitis. Among these 32 patients, a subgroup of 21 also underwent contrast-enhanced CT (CE-CT), which was used for a direct comparative analysis.

Given the absence of consensus on sample sizes for studies evaluating new diagnostic testing methods, the sample size could not be set based on statistical calculations. Instead, we enrolled as many cases as possible, referencing the prior literature [18,19,20]. The study protocol was developed and approved by the Ethics Committee of Nagoya City University Hospital (approval number: 60-22-0112) prior to data collection and analysis. This study was also registered at umin.ac.jp (UMIN000053798). The study adhered to the principles outlined in the Declaration of Helsinki. The requirement for written informed consent was waived due to the study’s retrospective nature. The full study protocol is available from the corresponding author upon request. The study adhered to the Standards for Reporting of Diagnostic Accuracy Studies (STARD) guidelines to ensure transparency and accuracy in reporting (Table S1).

### 2.2. Reference Standard

The final diagnosis of infectious aortitis, used as the reference standard, was established according to predefined diagnostic criteria that integrated clinical, radiological, and microbiological findings. Radiological findings were independently reviewed by two board-certified radiologists with expertise in vascular imaging to ensure diagnostic reliability. Since the American College of Cardiology Foundation/American Heart Association guidelines do not provide explicit diagnostic criteria for infectious aortitis, and although the MAGIC criteria are available for AGI, a unified standard for IAA has not yet been established [21]. To address this gap, we adapted the MAGIC criteria framework—originally validated for AGI—to encompass the entire spectrum of infectious aortitis. To enhance the robustness of this expanded application, we incorporated the core principles of the MAGIC criteria together with additional diagnostic elements derived from other key reports [2,22]. This comprehensive approach was designed to reflect real-world clinical decision-making where a definitive pathological diagnosis is not always available.

According to our study protocol, a definitive diagnosis was established if at least one major criterion or at least two minor criteria were met.

#### 2.2.1. Major Criteria

Surgical/Clinical Findings: Presence of pus around a graft or within an aneurysm sac observed during surgery (with microscopic confirmation); an open wound with an exposed graft, or the presence of a communicating sinus tract; fistula formation between the aorta/graft and another organ.

Imaging Findings: Perigraft fluid collection observed on CT scan more than 3 months after graft placement; perigraft gas observed on CT scan more than 7 weeks after graft placement; an increase in perivascular gas or fluid volume demonstrated on serial imaging studies.

Microbiological/Laboratory Findings: Isolation of a pathogen from a surgically resected aortic wall or graft specimen; isolation of a pathogen from intraoperative specimens (e.g., perivascular tissue, aneurysm contents).

#### 2.2.2. Minor Criteria

Clinical Findings: Local clinical features suggestive of infection (e.g., erythema, warmth, swelling, purulent discharge, pain); fever ≥ 38 °C where aortic infection is the most probable cause; shock (systolic blood pressure ≤ 80 mmHg) attributable to the aortic infection.

Imaging Findings: Other suspicious findings on imaging, such as suspicious perivascular gas, fluid, or soft-tissue inflammation; rapid expansion of the aneurysm; pseudoaneurysm formation; localized thickening of an adjacent bowel wall; or evidence of adjacent discitis or osteomyelitis.

Laboratory/Microbiological Findings: Positive blood cultures without another evident source of infection; markedly elevated inflammatory markers (e.g., C-reactive protein ≥ 5 mg/dL showing responsiveness to antibiotic therapy) where aortic infection is the most probable cause

### 2.3. Reference Standard for Sensitivity Analysis

To rigorously assess the diagnostic performance of the imaging modalities and mitigate potential incorporation bias from the inclusion of imaging findings in the composite reference standard, a sensitivity analysis was also performed. For this analysis, a stricter, imaging-independent reference standard was applied. A case was defined as having a definitive diagnosis of infectious aortitis only if it met the following criteria, based on surgical and microbiological evidence: positive culture from a surgically resected aortic wall or graft specimen, or direct observation and microscopic confirmation of pus during surgery, in a patient with concordant clinical signs of infection (e.g., fever, localized pain).

### 2.4. MRI Examinations

MRI was conducted using a 1.5-T scanner (MAGNETOM Avanto; Siemens, Erlangen, Germany) with phased-array spine and body matrix coils. No fasting was required before imaging. Patients were placed in a supine position and immobilized with the body coil in a head-first orientation. The imaging parameters for DWIBS, utilizing a Short Tau Inversion Recovery (STIR) sequence for fat suppression, were as follows: field of view (FOV), 450 mm; resolution, 132 × 132; number of slices, 40; slice thickness, 6 mm (gapless); repetition time (TR), 7000 ms; echo time (TE), 66 ms; inversion time (TI), 180 ms; number of averages, 5; b-value, 800 s/mm^2^; acquired under free-breathing; mode, parallel imaging technique (PAT) for generalized autocalibrating partially parallel acquisitions (GRAPPA2). Images were reconstructed using maximum intensity projection (MIP). For T2-weighted imaging with a half-Fourier acquisition single-shot turbo spin echo (HASTE) sequence, the imaging parameters were as follows: FOV, 450 mm; resolution, 256 × 154; number of slices, 40; slice thickness, 6 mm (gapless); TR, 600 ms; TE, 83 ms; breath-holding; and scan duration, 35 s. No contrast agents were administered before or during the imaging procedure. The MR unit calculated the apparent diffusion coefficient (ADC) map by selecting the abscess cavity.

### 2.5. CT Examinations

All CT examinations were performed on either a 256-multislice (SOMATOM Drive; Siemens, Erlangen, Germany) or a 128-multislice (SOMATOM Definition Flash; Siemens, Erlangen, Germany) CT system.

For the primary analysis in this study, CE-CT images were systematically evaluated. The acquisition parameters for these non-contrast scans were as follows: tube voltage, 100 kV; automated tube current modulation (CARE Dose 4D; Siemens) was used with a quality reference of 220 mAs at a reference tube voltage of 120 kV; slice thickness, 1 mm; pitch factor, 1.2; and FOV, 300–400 mm. Images were reconstructed using a soft-tissue kernel with noise reduction (Bf37).

For clinical indications, CE-CT was also performed in 21 patients. A weight-adapted protocol was utilized. Non-ionic iodinated contrast media (Iopamidol, Iopamiron, or Iopromide) with concentrations of 300–370 mgI/mL were administered, with volumes of 75–100 mL. The injection rate was 3.5–4.0 mL/s, followed by a 30 mL saline flush. Tube voltage was adjusted based on patient weight (90 kV for <70 kg, 100 kV for ≥70 kg). Arterial phase imaging was initiated 5 s after reaching a threshold of 120 Hounsfield Units (HU) in the terminal aorta, using a bolus-tracking technique. A delayed venous phase was acquired 70 s after the completion of the arterial phase scan.

### 2.6. Imaging Diagnosis

All imaging data were independently evaluated on a SYNAPSE VINCENT workstation (Version 6.4; Fujifilm Corporation, Tokyo, Japan) by two experienced radiologists who were blinded to clinical information. If the two radiologists’ opinions differed, they reached a consensus through a final qualitative evaluation by discussion, a method previously reported for qualitative imaging assessments [10,23].

The evaluation process was structured as follows:

#### 2.6.1. Primary Analysis (Full Cohort, *n* = 32)

The primary analysis involved the evaluation of NCCT and DWIBS images.

On NCCT, a positive diagnosis was based on established morphological features such as periaortic soft tissue stranding, periaortic gas, and rapid aortic expansion [2]. Additionally, for patients with aortic grafts, perigraft swelling, fluid, or gas were also considered positive findings [21]. Due to the complexity of these findings, quantitative evaluation using the HU was not performed.

DWIBS was considered positive when an area of higher signal intensity was observed in the enhanced soft tissue within or around the aorta than that of the reference object [24,25,26]. The signal intensities in the spinal cord (DWIBS [spinal cord]) and the normal aorta (DWIBS [aorta]) were used as references. As an exploratory analysis, the ADC value could not be measured in the absence of an abscess cavity; therefore, measurements were only taken in cases where it was possible [27].

A positive diagnosis for the combined DWIBS/NCCT model required positive findings on both NCCT and DWIBS.

#### 2.6.2. Subgroup Analysis (*n* = 21)

For the subgroup of 21 patients who underwent CE-CT, a comprehensive diagnostic judgment was made. This process began with an assessment of the NCCT series for the findings described above. Subsequently, the contrast-enhanced series was evaluated for additional, contrast-dependent findings. A final comprehensive judgment was made based on the presence of features such as saccular or multilobulated aneurysm shape, aortic wall enhancement, fistula, or abscess formation [2,21].

### 2.7. Statistical Analysis

All analyses conducted in this study were based on a strict protocol defined before data collection commenced. Continuous variables were considered non-normally distributed and were presented as medians (with interquartile ranges), and categorical variables are represented as frequencies and percentages. Values are rounded to appropriate decimal places for clinical relevance. The diagnostic performance of different imaging models was assessed for both the full cohort (*n* = 32) and a subgroup of patients who underwent all imaging modalities (*n* = 21). Sensitivity, specificity, and positive and negative predictive values are presented with corresponding 95% confidence intervals (CIs). Receiver operating characteristic (ROC) curve, areas under the curve (AUC) were calculated. For the DWIBS ADC values, logistic regression models were created to calculate ROC curves, AUCs. The sensitivity and specificity, along with their corresponding 95% CIs, were then determined by establishing cutoff values. We also calculated effect estimates with their 95% CIs to assess the diagnostic parameters and performed DeLong’s test for each ROC curve. We then assessed the inter-reader reliability between the two readers using Cohen’s kappa coefficient for binary values and intraclass correlation coefficients for continuous quantities. Logistic regression and decision curve analysis (DCA) were performed to assess the potential benefit of adding DWIBS to NCCT. A logistic regression model was constructed using DWIBS and NCCT as independent variables. The AUC was calculated for the model, and the difference between the DWIBS + NCCT model and the NCCT-alone model was compared using DeLong’s test. DCA was conducted to evaluate the comparative clinical utility of the DWIBS + NCCT model versus the NCCT-alone model across a range of threshold probabilities.

In the DCA analysis, a net benefit of clinical judgment to opt or not opt for the treatment based on a prediction model was assessed by comparing harm (treating a patient without disease) to benefit (treating a patient with disease). The harm-to-benefit ratio corresponds to a threshold probability where the expected benefit of treatment equals the expected benefit of avoiding treatment [28]. In the opt-in policy, the net benefit of receiving treatment was calculated using true positives and false positives, resulting in a higher net benefit in the setting of a smaller harm-to-benefit ratio. Conversely, under the opt-out policy, the net benefit of avoiding treatment was derived from true negatives and false negatives and was higher when the harm-to-benefit ratio became greater. Net benefits were graphically represented against threshold probabilities to assess the clinical value of the models [28,29,30].

Statistical analyses were performed using R (version 4.2.2; R Foundation for Statistical Computing, Vienna, Austria), with the “rmda” package utilized for DCA. *p*-values ≤ 0.05 were considered statistically significant (two-tailed). Analyses involving ADC values were performed only on the subset of patients for whom these measurements were available.

## 3. Results

### 3.1. Characteristics of the Patients

Table 1 presents the baseline characteristics of the patients, stratified by the final diagnosis of infectious or non-infectious aortitis. The median age was 76 years (interquartile range, 46–94 years), and 9 (28%) were female. Fifteen (47%) patients were diagnosed with infectious aortitis, including nine (28%) with IAA and six (40%) with AGI. Among those diagnosed with infectious aortitis, fever was noted in 15 (100%), pain in 10 (67%), and positive blood culture in 11 (73%) patients. The diagnoses of 17 (53%) patients without infectious aortitis included non-IAA in three, acute aortic dissections in two, infectious endocarditis in three, superior mesenteric artery dissections in two, postoperative wound infections in two, giant cell arteritis in one, and spondylitis in one patient. Detailed characteristics of all 32 patients, including whether CE-CT was performed, are provided in Table S2. The ADC values were measurable in 19 cases. Of these, 12 were in the infectious aortitis group and 7 were in the non-infectious group.

Among patients with infectious aortitis, eight (53%) underwent surgical treatment, including prosthetic graft replacement and stent grafting in five (33%) and four (27%) patients, respectively. One patient underwent stent grafting followed by prosthetic graft replacement as a planned two-stage surgery. One patient required an unplanned second open surgery after stent grafting due to reinfection. Another patient died from sepsis due to reinfection after stent grafting. The other six patients had no major complications. In contrast, seven (47%) patients who did not undergo surgery, including stent grafting, were treated with antibiotics owing to poor condition or patient refusal. Among these, four patients died from aortic infection-related events: sepsis in three and aortic rupture in one patient. No MRI (including DWIBS)-associated adverse events, such as dizziness or nausea after the test, were observed.

### 3.2. Diagnostic Performance of DWIBS and CT

#### 3.2.1. Primary Analysis in the Full Cohort (*n* = 32)

In the qualitative assessment of DWIBS, the choice of reference significantly impacted diagnostic performance, as summarized in Table 2. Using the spinal cord as a reference resulted in 18 (56%) positive cases, with a sensitivity of 93.3%, specificity of 76.5%, and an AUC of 0.85. In contrast, using the aorta as a reference yielded 23 (72%) positive cases, with a lower specificity of 47.1% and an AUC of 0.70. The inter-reader agreement for this qualitative assessment was high, with kappa coefficients of 0.88 for DWIBS (spinal cord) and 0.86 for DWIBS (aorta).

NCCT alone demonstrated good diagnostic performance, but the combination with DWIBS (spinal cord) yielded higher diagnostic performance. NCCT alone was positive in 19 patients, with a sensitivity of 93.3%, specificity of 70.6%, and an AUC of 0.82 (Table S4). When combined with NCCT, the DWIBS (spinal cord) model yielded a sensitivity of 86.7%, a specificity of 94.1%, and an AUC of 0.90. In comparison, the DWIBS (aorta)+NCCT model showed a lower specificity of 70.6% and an AUC of 0.79. The AUC for the DWIBS (spinal cord)+NCCT model was significantly higher than that for the DWIBS (aorta)+NCCT model (difference 11.8%; 95% CI: 1.37–22.16%; *p* = 0.027), demonstrating its higher diagnostic utility in the primary analysis.

We further evaluated diagnostic performance stratified by aortic pathology and postoperative phase (Table S5). In IAA (*n* = 15), sensitivity was 88.9% and specificity was 100%. In AGI (*n* = 17), sensitivity was 100% and specificity was 63.6%. Excluding the early postoperative phase (<3 months) resulted in a specificity of 83.3%.

For the quantitative analysis using ADC values, which were measurable in 19 cases, the results were less clinically applicable. While the AUCs for reader 1 and reader 2 were 0.77 and 0.81, respectively, applying a cutoff value resulted in poor diagnostic performance, with sensitivities of 91.7% and 75% but specificities of 0% for both readers (Table S3 and Figure S1). The inter-reader reliability for ADC measurements was excellent, with an intraclass correlation coefficient of 0.88.

#### 3.2.2. Sensitivity Analysis with a Strict Reference Standard

The sensitivity analysis was conducted on a cohort of 32 patients, comprising 8 with surgically and microbiologically confirmed infectious aortitis and 24 confirmed as negative by the same strict, imaging-independent criteria. The diagnostic performance of each modality in this cohort is summarized in Table 3.

The combined DWIBS (spinal cord) + NCCT model achieved the highest specificity of 70.8% of the three modalities. Regarding overall diagnostic performance, the AUCs for both the combined model and DWIBS alone were 0.79, which was higher than the AUC of 0.69 for NCCT alone.

#### 3.2.3. Subgroup Analysis (*n* = 21)

A subgroup analysis was performed on the 21 patients who also underwent CE-CT. The sensitivity, specificity, AUC of CE-CT were 100.0%, 30.0%, 0.65. The inter-reader agreement was assessed using kappa coefficients, which were 0.83 for CE-CT. The AUC for the combined DWIBS (spinal cord)+NCCT diagnosis, 0.90, was significantly higher than that for CE-CT, 0.65 (difference 25.4%; 95% CI: 9.3–41.4%; *p* = 0.0019) in the subgroup. The detailed diagnostic performance for each model within this subgroup is presented in Table 4.

### 3.3. Diagnostic Model

**Figure 2:**
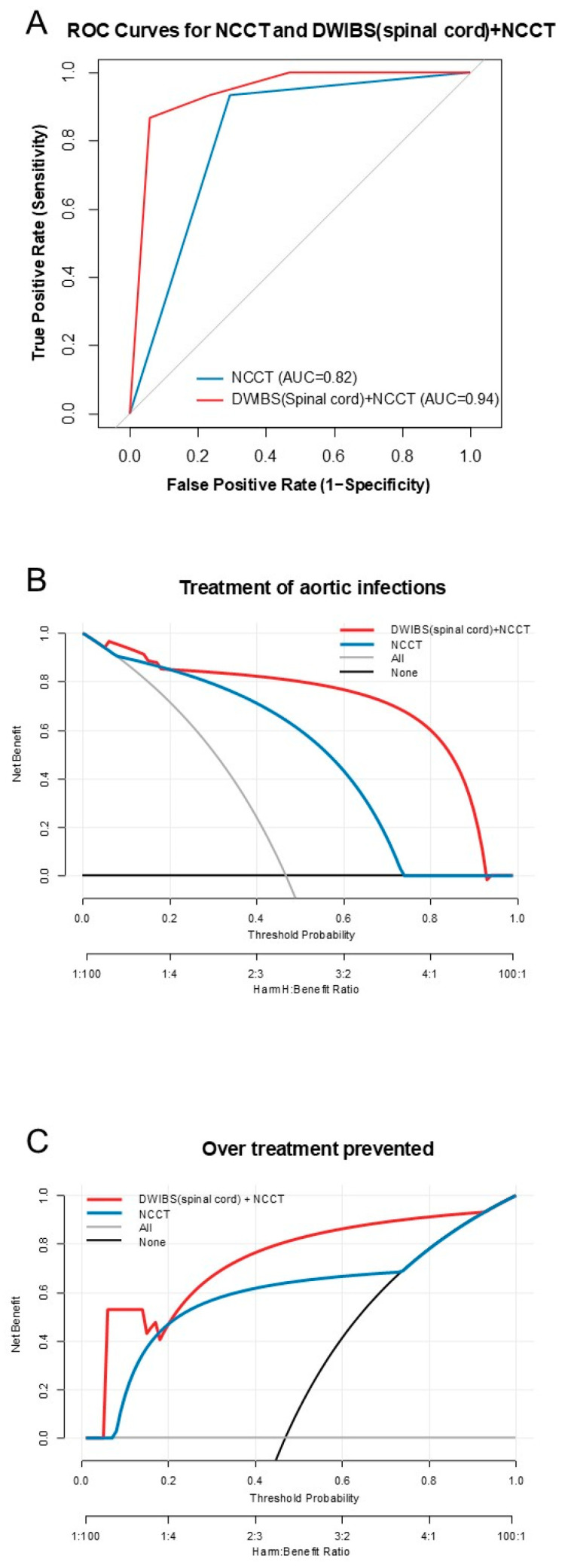
Comparison of diagnostic performance between DWIBS and NCCT. (**A**) ROC curves for predicting infectious aortitis using NCCT alone and the combination of DWIBS (spinal cord) and NCCT. (**B**) Net benefits of receiving treatment for infectious aortitis based on the NCCT alone model and the DWIBS + NCCT model. (**C**) Net benefits of avoiding treatment based on each model. Net benefits when all patients received or avoided treatment are also given. NCCT, non-contrast computed tomography; DWIBS, diffusion-weighted whole-body imaging with background suppression; ROC, receiver operating characteristic.

To explore the added value of DWIBS, a logistic regression model incorporating both DWIBS and NCCT was constructed. The AUC for DWIBS (spinal cord) + NCCT was 0.94 (95% CI: 0.86–0.10), which tended to be higher than the AUC for NCCT alone (difference 12.1%, 95% CI: −0.29–24.60%; *p* = 0.056; Figure 2A).

The DCA analysis demonstrated that the DWIBS + NCCT model provided a higher net benefit to the NCCT-alone model on almost all threshold probabilities in both opt-in and opt-out policies. The net benefit of receiving treatment was higher in the DWIBS + NCCT model than in other models, especially at a threshold probability of greater than 20% (Figure 2B). In contrast, the net benefit of avoiding treatment was higher in the DWIBS + NCCT model than in the others at all threshold probabilities (Figure 2C). The comparative ROC curve and DCA for the three diagnostic strategies in the subgroup analysis is shown in Figure S2.

## 4. Discussion

To our knowledge, this exploratory study is the first to evaluate DWIBS (spinal cord) for infectious aortitis. Our primary analysis (*n* = 32) used a composite reference standard that included imaging findings, which carries a risk of incorporation bias. To address this, we performed a sensitivity analysis using a strict, imaging-independent standard based only on surgical or microbiological proof. This sensitivity analysis (Table 3) provided a crucial insight: the trend observed in the primary analysis (Table 2) was replicated. Specifically, adding DWIBS to NCCT improved the overall diagnostic performance (AUC: 0.79 vs. 0.69) over NCCT alone, just as it did in the primary analysis (AUC: 0.90 vs. 0.82). This consistency across both analyses, despite the different reference standards, supports the robustness of our main conclusion.

The utility of PET/CT and MRI/MRA for the diagnosis of inflammatory aortitis, which includes infectious aortitis, is widely recognized. In a large cohort of patients, for instance, the sensitivity for detecting the disease was reported to be 91.4% for PET/CT and 83.3% for MRI/MRA [31]. However, the diagnostic performance varies depending on the specific etiology. When focusing on infectious aortitis, the specificity of PET/CT has been reported at 57.1% for IAA [19] and 83.3% for AGI when combined with CE-CT [32], indicating room for improvement. For context, in suspected vascular graft/endograft infection, the pooled sensitivity/specificity were reported as 67.0%/63.0% for CTA and 95.0%/80.0% for PET/CT [33]. To address these limitations, novel radiotracers targeting somatostatin receptor 2 on activated macrophages or specific bacteria are currently under development [34,35,36]. In contrast, reports on the use of MRI for diagnosing infectious aortitis remain limited, although recent studies have highlighted its potential for evaluating aortic pathologies such as endoleaks and inflammation [37,38,39]. In this context, the combined DWIBS and NCCT approach used in our institution demonstrated a high specificity of 94.1% in the primary analysis.

Our findings suggest that the protocols and positive criteria of DWIBS for infectious aortitis may need to differ from those generally employed for tumor screening. Despite the numerous quantitative assessments used in cancer diagnosis that utilize ADC values, the non-uniform nature of fluid components in abscess cavities and infected organs likely affects the reproducibility of ADC values. A high signal area may not exist without an abscess lumen, making quantitative evaluation challenging. Among the 19 cases in which ADC values could be measured, the AUC of the logistic regression model was equivalent to that of the qualitative evaluation; however, the diagnostic performance with a cutoff value was lower, suggesting challenges in its practical application within clinical settings. Furthermore, although qualitative evaluations are often made by comparing the same tissue, the aorta is luminal and contains few parenchymal components. Additionally, the infection can spread over the entire circumference, making it inadvisable to target the same organ. For these reasons, we considered that qualitative evaluation of DWIBS, achieved by comparing spinal cord and signal values, was useful. DWIBS (spinal cord) demonstrated strong reproducibility with minimal inter-reader variability. Therefore, comparison with the spinal cord, an organ located within the imaging range and containing fluid components, appears to be a simple and effective approach.

A key strength of our study was the combination of DWIBS with NCCT. In our subgroup analysis, we found that although NCCT and CE-CT had comparable sensitivity, CE-CT produced more false-positive findings. Notably, the three postoperative cases yielded false positives only on the CE-CT assessment. In the postoperative setting, normal healing can involve perigraft fluid and small amounts of gas that may persist for several months [39,40], making differentiation from a true infection extremely challenging.

Other false positives were shared by both modalities, including intramural hematomas from dissections (*n* = 3) and Giant Cell Arteritis (*n* = 1). This reflects the known challenge that findings such as aortic wall enhancement are not specific to infection [33,41,42]. Despite these challenges, however, CE-CT remains an essential tool [43,44]. In our study, it was effective in correctly diagnosing a saccular aneurysm that was a false positive on NCCT alone, and its high inter-reader agreement in the subgroup suggests its stability as an objective modality. It is precisely because conventional CT modalities have these distinct diagnostic limitations that the functional information provided by DWIBS offers complementary value, thereby improving diagnostic accuracy. Consequently, DWIBS may serve as a potential non-contrast alternative, particularly for patients with renal insufficiency or contrast allergy, or in settings where PET/CT is unavailable.

**Figure 3:**
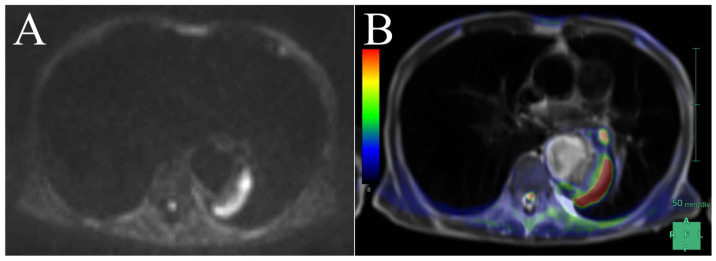
Additive composition of DWIBS and T2-weighted images. (**A**) Axial DWIBS imaging (b = 800 s/mm^2^, slice thickness: 6 mm) showing high signal intensity in the periaortic abscess. (**B**) A composite of axial DWIBS and T2-weighted HASTE images is shown; the combination of DWIBS with pseudo-colored T2-weighted images produces a composite image resembling PET/CT. The fusion ratio was adjusted to optimize the visualization of both anatomical structures and inflammatory signals. Window settings were optimized for soft-tissue contrast to delineate the abscess. DWIBS signal intensity is displayed in pseudo-color, where red indicates high signal intensity and blue indicates lower signal intensity. DWIBS, diffusion-weighted whole-body imaging with background suppression; HASTE, Half-Fourier Acquisition Single-shot Turbo spin Echo; CT, computed tomography; PET, positron emission tomography.

**Figure 4:**
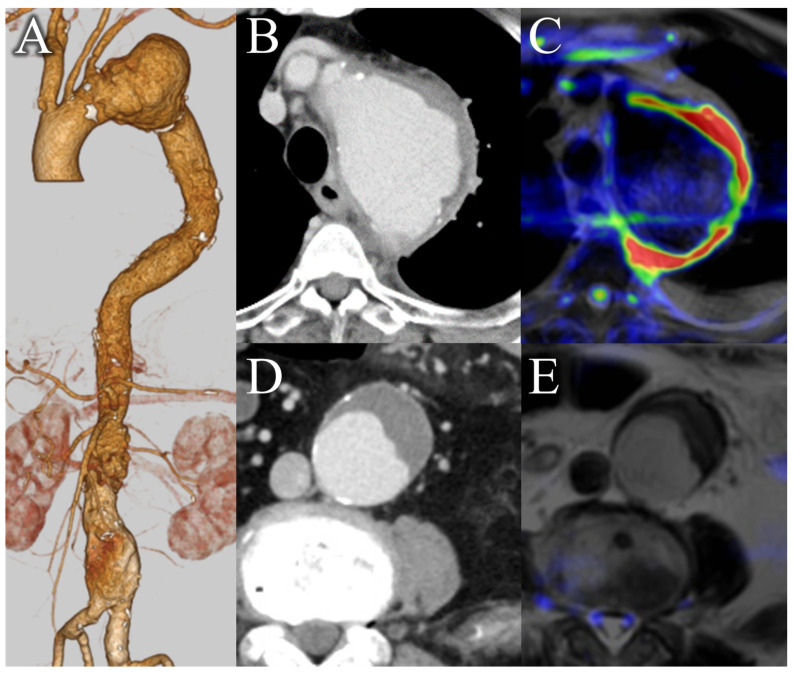
Identifying the extent of infection using DWIBS. A case of aortic aneurysms in both the thoracic and abdominal regions. (**A**) A 3D reconstruction using CE-CT, (**B**) Axial CE-CT of the thorax, (**C**) axial thoracic DWIBS (b = 800 s/mm^2^) fused with T2-weighted imaging, (**D**) axial CE-CT of the abdomen, and (**E**) axial abdominal DWIBS fused with T2-weighted imaging. In the fused images (**C**,**E**), the blending ratio of DWIBS to T2-weighted images was adjusted to ensure accurate localization. In (**B**), mild enhancement of the soft-tissue shadow around the aorta, along with a mural thrombus, is noted; the DWIBS image in (**C**) shows high signal intensity in the same area. In (**D**), no soft-tissue shadow is visible, although a large mural thrombus is present. In (**E**), no high signal intensity is observed. The findings indicate that the infection is likely confined to the thoracic aortic aneurysm. CT images are displayed with standard soft-tissue window settings. DWIBS signal intensity is displayed in pseudo-color, where red indicates high signal intensity and blue indicates lower signal intensity. DWIBS, diffusion-weighted whole-body imaging with background suppression; CE-CT, contrast-enhanced computed tomography; CT, computed tomography.

In the present study, the b-value was set to 800 s/mm^2^. Although a b-value of 1000 s/mm^2^ is often used for tumor screening, we aimed to evaluate both the abscess cavity and tissue edema due to infection in the context of aortic infections, suggesting the utility of a high-resolution approach [26]. Higher b-values might reduce the T2 effect, making it potentially challenging to identify tissue edema and potentially increasing the likelihood of false negatives [45]. Additionally, fibrotic or calcified plaques might exhibit low signal intensity, potentially obscuring the surrounding inflammation on T2-weighted images [46]. This phenomenon could explain the false-negative case observed in our study. In our MRI protocol, we employed HASTE for T2-weighted imaging. HASTE can be rapidly performed and clearly delineates anatomical structures. By applying pseudo-color to DWIBS on a workstation and then fusing it with HASTE images, we can simulate PET/CT-like images and visualize the extent of infection (Figure 3) [47]. Additionally, even in cases involving overlapping aortic aneurysms, the extent of infection can be clearly identified, which is helpful for surgical planning (Figure 4). However, T2-weighted imaging is not useful for differentiation because both subacute hematomas and abscesses exhibit high signal intensity, similar to DWIBS.

Infectious aortitis is associated with relatively greater harm when treating patients without disease, as the radical treatment of this entity is surgical and usually highly invasive. Therefore, an exploratory DCA analysis is quite effective, as it provides a preliminary view of the net benefit of clinical judgment based on each diagnostic modality over a range of harm-to-benefit ratios. In this study, the DCA analysis supported the potential efficacy of adding DWIBS to NCCT to diagnose infectious aortitis. In an opt-in approach, a certain level of net benefit was obtained with NCCT alone at a low threshold probability; however, a higher net benefit was obtained by adding DWIBS, especially in cases with a higher harm-to-benefit ratio. This suggested that while a diagnosis using NCCT alone might be sufficiently beneficial to initiate less invasive treatment, such as medication with antibiotics, adding DWIBS appears more effective for decision-making to opt for more invasive treatment. In contrast, an opt-out approach using DWIBS with NCCT also showed a greater net benefit of avoiding treatment than NCCT alone. However, the difference between them was relatively constant throughout all threshold probabilities. These findings suggest that the addition of DWIBS to NCCT is beneficial in the clinical decision-making process for aortic infections, particularly in determining the need for invasive treatments. Therefore, in future prospective studies, it is desirable to validate the utility within high threshold ranges.

**Figure 5:**
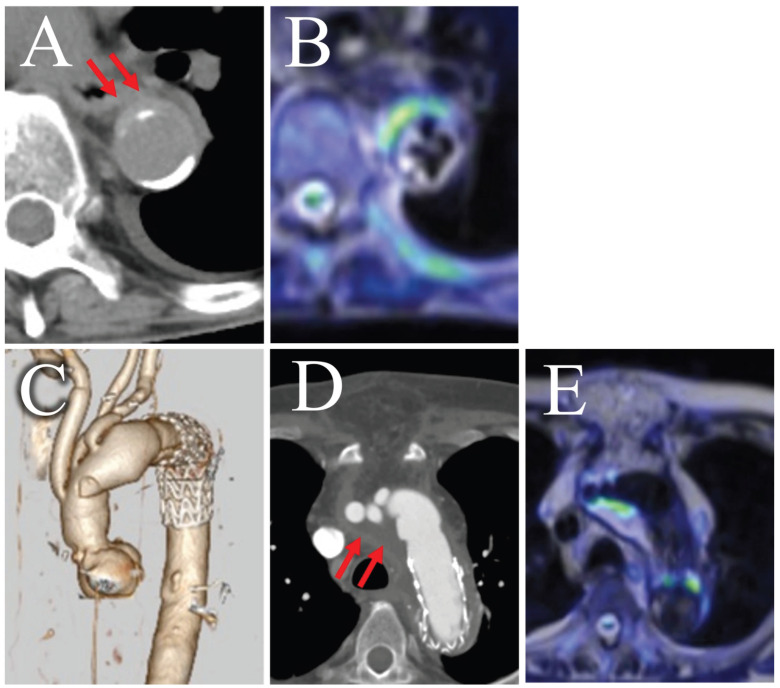
Pitfalls of DWIBS. Accurate diagnosis requires integrating multiple imaging modalities. Relying solely on MRI, especially DWIBS and T2-weighted imaging, may not suffice for comprehensive assessment. (**A**,**B**) A case of a thrombosed aortic dissection. (**A**) Axial NCCT (soft-tissue window); (**B**) axial DWIBS (b = 800 s/mm^2^) fused with T2-weighted imaging. In (**A**), the false lumen (arrows) shows high attenuation on NCCT, whereas in (**B**), it appears hyperintense on DWIBS. Differentiating these features using DWIBS alone is challenging, but CT values facilitate an accurate distinction. (**C**–**E**) Early postoperative images following surgical repair of an infectious aortic aneurysm. (**C**) A 3D reconstruction from CE-CT; (**D**) an axial CE-CT image that shows no enhancement around the graft but reveals a soft-tissue shadow (arrow); (**E**) DWIBS fused with T2-weighted imaging, demonstrating partially high signal intensity around the graft. However, based on clinical findings and follow-up, no evidence of infection was found. Detailed imaging parameters: DWIBS (b = 800 s/mm^2^, slice thickness: 6 mm); CT (slice thickness: 1 mm, soft-tissue kernel). In the fused images (**B**,**E**), the blending ratio of DWIBS to T2-weighted images was adjusted to ensure accurate localization. DWIBS signal intensity is displayed in pseudo-color, where red indicates high signal intensity and blue indicates lower signal intensity. DWIBS, diffusion-weighted whole-body imaging with background suppression; MRI, magnetic resonance imaging; NCCT, non-contrast computed tomography; CE-CT, contrast-enhanced computed tomography; CT, computed tomography.

This study had some limitations. First, as this was a retrospective, single-center study, selection bias cannot be excluded. Second, while our primary diagnostic model was based on a composite reference standard, we attempted to validate its findings using a stricter, imaging-independent standard. However, this sensitivity analysis was limited by the small number of patients who met these strict criteria. As noted in the results, only 9 patients underwent surgical treatment, which was the primary source for the strict criteria, resulting in a positive cohort of only 8 patients. Although the results showed a consistent trend, the precise estimates of sensitivity and specificity in this subgroup must be interpreted with caution. Given the small sample size and resulting wide confidence intervals across all groups, this study should be considered preliminary and hypothesis-generating. Prospective validation in larger cohorts is necessary to confirm these findings across the full spectrum of disease severity. Third, a comparison with contrast-enhanced MRI was not performed as it is not a routine examination for this condition at our institution. Fourth, the decision curve analysis, while illustrative, must be interpreted with caution as it is inherently hypothetical in a retrospective framework and serves primarily to generate hypotheses for future prospective trials. Fifth, although IAA and AGI are distinct, we pooled them based on their shared ‘diagnostic decision point’ in clinical practice [4,48,49,50,51]. Our stratified analysis (Table S5) confirms that DWIBS maintains high diagnostic value for both IAA and chronic AGI. Nonetheless, the criteria for suspicion were clearly defined, and consecutive cases were included in the study. Furthermore, differentiating infectious aortitis from acute thrombosed aortic dissection can be challenging, as both conditions exhibit high signal intensity on DWI. False-positive results may also arise in the early postoperative period following prosthetic graft replacement, where perigraft fluid accumulation often presents with high signal intensity, similar to the challenges observed in CE-CT. Subacute hematomas typically exhibit higher attenuation on CT, while abscess cavities appear with low signal intensity, which helps differentiate them from thrombotic aortic dissections. However, differentiating inflammatory lesions from postoperative changes remains a challenge even with multiparametric MRI (Figure 5). Adding various MRI sequences and follow-up imaging may improve diagnostic accuracy in the future. Therefore, the DWIBS-based approach currently serves as a complementary tool rather than a standalone replacement.

## 5. Conclusions

The results of this exploratory study suggest that the combination of NCCT and DWIBS, a previously underutilized MRI technique, offers a promising diagnostic approach for patients with suspected infectious aortitis. This combination may be particularly valuable for patients in whom contrast-enhanced imaging is contraindicated. Further validation in larger, multicenter prospective studies is warranted to confirm these preliminary findings.

## Figures and Tables

**Table 1:** Baseline characteristics of the patients with suspected infectious aortitis.

Characteristic	Overall *N* = 32	Infectious Aortitis *N* = 15	Non-Infectious Aortitis *N* = 17
Age, years ^a^	76 (64, 83)	81 (74, 87)	70 (61, 80)
Male sex, *n*	23 (72%)	9 (60%)	14 (82%)
Height, cm ^a^	165 (158, 167)	162 (152, 165)	166 (164, 167)
Weight, kg ^a^	54.6 (48.7, 58.8)	50.0 (45.1, 60.3)	58.0 (52.0, 58.7)
Body mass index, kg/m^2 a^	20.8 (19.3, 22.2)	19.6 (18.7, 23.8)	21.0 (20.4, 21.1)
Pain, *n*	19 (59%)	10 (67%)	9 (53%)
Fever, *n*	23 (72%)	15 (100%)	8 (47%)
Shock, *n*	2 (6.2%)	1 (6.7%)	1 (5.9%)
Culture positive, *n*	17 (53%)	10 (67%)	7 (41%)
C-reactive protein ^a^	14.8 (9.1, 21.8)	17.3 (14.3, 24.1)	10.4 (6.5, 18.8)
Leukocyte count, 10^3^/μL ^a^	9.80 (6.33, 12.4)	12.1 (11.4, 13.8)	6.40 (4.60, 9.60)
Procalcitonin, ng/mL ^a^	0.27 (0.06, 1.67)	0.51 (0.17, 1.54)	0.12 (0.03, 1.57)
Ascending, *n*	3 (9.4%)	0 (0%)	3 (18%)
Arch, *n*	15 (47%)	9 (60%)	6 (35%)
Descending, *n*	5 (16%)	3 (20%)	2 (12%)
Paravisceral, *n*	4 (12%)	1 (7%)	3 (18%)
Infrarenal, *n*	4 (12%)	2 (13%)	2 (12%)
Iliac, *n*	2 (6.2%)	2 (13%)	0 (0%)
Multiple, *n*	4 (12%)	3 (20%)	1 (6%)
Hypertension, *n*	15 (47%)	9 (60%)	6 (35%)
Diabetes mellitus, *n*	5 (16%)	4 (27%)	1 (5.9%)
Lipid disorders, *n*	8 (25%)	4 (27%)	4 (24%)
Chronic kidney disorder, *n*	6 (19%)	3 (20%)	3 (18%)
Cancer, *n*	3 (9%)	2 (13%)	1 (6%)
Post-aortic surgery, *n*	17 (53%)	6 (40%)	11 (65%)
Within 3 months, *n*	5 (16%)	0 (0%)	5 (29%)
Open surgery, *n*	9 (28%)	5 (33%)	4 (24%)
Endovascular surgery, *n*	4 (12%)	4 (27%)	1 (6%)

*n* (%); ^a^ Median (interquartile range).

**Table 2:** Diagnostic performance of each imaging modality.

	TP	FP	TN	FN	Sensitivity	Specificity	PPV	NPV	AUC
DWIBS (spinal cord) ^a^	14	4	13	1	93.3 (68.1–99.8)	76.5 (50.1–93.2)	77.8 (52.4–93.6)	92.9 (66.1–99.8)	0.85 (0.73–0.97)
DWIBS (aorta) ^b^	14	9	8	1	93.3 (68.1–99.8)	47.1 (23.0–72.2)	60.9 (38.5–80.3)	88.9 (51.8–99.7)	0.70 (0.56–0.84)
NCCT	14	5	12	1	93.3 (68.1–99.8)	70.6 (44.0–89.7)	73.7 (48.8–90.9)	92.3 (64.0–99.8)	0.82 (0.69–0.95)
DWIBS (spinal cord) + NCCT ^a^	13	1	16	2	86.7 (59.5–98.3)	94.1 (71.3–99.9)	92.9 (66.1–99.8)	88.9 (65.3–98.6)	0.90 (0.80–1.00)
DWIBS (aorta) + NCCT ^b^	13	5	12	2	86.7 (59.5–98.3)	70.6 (44.0–89.7)	72.2 (46.5–90.3)	85.7 (57.2–98.2)	0.79 (0.64–0.93)

*n*, % (% confidence interval); Abbreviations: TP, true positive; FP, false positive; TN, true negative; PPV, positive predictive value; NPV, negative predictive value; AUC, area under the curve; DWIBS, diffusion-weighted whole-body imaging with background suppression; NCCT, non-contrast computed tomography. ^a^ spinal cord was used as a reference. ^b^ aorta was used as a reference.

**Table 3:** Diagnostic Performance in Sensitivity Analysis Using a Strict, Imaging-Independent Reference Standard.

	TP	FP	TN	FN	Sensitivity	Specificity	PPV	NPV	AUC
NCCT	7	12	12	1	87.5 (47.3–99.7)	50.0 (29.1–70.9)	36.8 (16.3–61.6)	92.3 (64.0–99.8)	0.68 (0.53–0.85)
DWIBS (spinal cord) ^a^	8	10	14	0	100.0 (63.1–100.0)	58.3 (36.6–77.9)	44.4 (21.5–69.2)	100.0 (76.8–100.0)	0.79 (0.64–0.95)
DWIBS (spinal cord) + CT ^a^	7	7	17	1	87.5 (47.3–99.7)	70.8 (48.9–87.4)	50.0 (23.0–77.0)	94.4 (72.7–99.9)	0.79 (0.69–0.89)

*n*, % (% confidence interval); Abbreviations: TP, true positive; FP, false positive; TN, true negative; PPV, positive predictive value; NPV, negative predictive value; AUC, area under the curve; DWIBS, diffusion-weighted whole-body imaging with background suppression; CT, computed tomography. ^a^ spinal cord was used as a reference.

**Table 4:** Performance of Each Diagnostic Strategy in the Subgroup with Complete Imaging.

	TP	FP	TN	FN	Sensitivity	Specificity	PPV	NPV	AUC
NCCT alone	11	4	6	0	100 (71.5–100)	60.0 (26.2–87.8)	73.3 (44.9–92.2)	100 (54.1–100)	0.80 (0.64–0.96)
NCCT + CE-CT	11	7	3	0	100 (71.5–100)	30.0 (6.7–65.2)	61.1 (35.7–82.7)	100 (29.2–100)	0.65 (0.50–0.80)
DWIBS (spinal cord) ^a^ + NCCT	10	1	9	1	90.9 (58.7–99.8)	90.0 (55.5–99.7)	90.9 (58.7–99.8)	90.0 (55.5–99.7)	0.93 (0.82–1.00)
DWIBS (spinal cord) ^a^ + NCCT + CE-CT	10	3	7	1	90.9 (58.7–99.8)	70.0 (34.8–93.3)	76.9 (46.2–95.0)	87.5 (47.3–99.7)	0.80 (0.64–0.96)

*n*, % (% confidence interval); Abbreviations: TP, true positive; FP, false positive; TN, true negative; PPV, positive predictive value; NPV, negative predictive value; AUC, area under the curve; ADC, apparent diffusion coefficient; NCCT, non-contrast computed tomography, CE-CT; contrast enhanced computed tomography; DWIBS, diffusion-weighted whole-body imaging with background suppression; ^a^ spinal cord was used as a reference.

## Data Availability

The data presented in this study are available on request from the corresponding author due to privacy and ethical restrictions.

## Supplementary Materials

The following supporting information can be downloaded at: https://www.mdpi.com/article/10.3390/diagnostics16020225/s1, Figure S1: AUC curves for each reader when using ADC values; Figure S2: Comparison of diagnostic performance in subgroup, Table S1: STARD 2015 checklist; Table S2: Characteristics of cases enrolled due to suspected infectious aortitis; Table S3: Comparison of each evaluation method in diffusion-weighted whole-body imaging with background body signal suppression; Table S4: Supplement to the character baseline: Reading findings by readers 1 and 2. Table S5: Diagnostic performance of DWIBS and NCCT stratified by aortic pathology and postoperative phase.

## Abbreviations

The following abbreviations are used in this manuscript:

DWIBS	Diffusion-Weighted Whole-Body Imaging with Background Body Signal Suppression
CT	Computed Tomography
HASTE	Half-Fourier Acquisition Single-shot Turbo spin Echo
ADC	Apparent diffusion coefficient
