# Supplementary material for: Diffusion-Weighted Whole-Body Magnetic Resonance Imaging with Background Body Signal Suppression for Differentiating Infectious from Non-Infectious Aortitis
Source: Diagnostics (Basel). 2026 Jan 10;16(2):225. doi: 10.3390/diagnostics16020225 (PMC12839786; doi:10.3390/diagnostics16020225)

Supplemental Material

Table S1. STARD 2015 checklist

Section & Topic	No	Item	Reported on page #
TITLE OR ABSTRACT			
	1	Identification as a study of diagnostic accuracy using at least one measure of accuracy<br>(such as sensitivity, specificity, predictive values, or AUC)	Title and Abstract
ABSTRACT			
	2	Structured summary of study design, methods, results, and conclusions (for specific guidance, see STARD for Abstracts)	Abstract
INTRODUCTION			
	3	Scientific and clinical background, including the intended use and clinical role of the index test	Introduction
	4	Study objectives and hypotheses	Introduction
METHODS			
Study design	5	Whether data collection was planned before the index test and reference standard<br>were performed (prospective study) or after (retrospective study)	Section 2.1 Study design
Participants	6	Eligibility criteria	Section 2.1 Study design
	7	On what basis potentially eligible participants were identified (such as symptoms, results from previous tests, inclusion in registry)	Section 2.1 Study design
	8	Where and when potentially eligible participants were identified (setting, location and dates)	Section 2.1 Study design
	9	Whether participants formed a consecutive, random or convenience series	Section 2.1 Study design
Test methods	10a	Index test, in sufficient detail to allow replication	Sections 2.3 MRI examinations and 2.4 CT examinations
	10b	Reference standard, in sufficient detail to allow replication	Section 2.2 Clinical diagnosis
	11	Rationale for choosing the reference standard (if alternatives exist)	Section 2.2 Clinical diagnosis
	12a	Definition of and rationale for test positivity cut-offs or result categories of the index test, distinguishing pre-specified from exploratory	Section 2.5 Imaging diagnosis
	12b	Definition of and rationale for test positivity cut-offs or result categories of the reference standard, distinguishing pre-specified from exploratory	Section 2.2 Clinical diagnosis
	13a	Whether clinical information and reference standard results were available	Section 2.5 Imaging diagnosis

		to the performers/readers of the index test	
	13b	Whether clinical information and index test results were available to the assessors of the reference standard	Section 2.2 Clinical diagnosis
Analysis	14	Methods for estimating or comparing measures of diagnostic accuracy	Section 2.6 Statistical analysis
	15	How indeterminate index test or reference standard results were handled	Section 2.5 Imaging diagnosis
	16	How missing data on the index test and reference standard were handled	Section 2.6 Statistical analysis
	17	Any analyses of variability in diagnostic accuracy, distinguishing pre-specified from exploratory	Section 2.6 Statistical analysis
	18	Intended sample size and how it was determined	Section 2.1 Study design
RESULTS			
Participants	19	Flow of participants, using a diagram	Section 2.1 Study design, Figure 1
	20	Baseline demographic and clinical characteristics of participants	Section 3.1 Patient Characteristics of the patients, Table 1
	21a	Distribution of severity of disease in those with the target condition	Section 3.1 Characteristics of the patients
	21b	Distribution of alternative diagnoses in those without the target condition	Section 3.1 Characteristics of the patients, Table S2
	22	Time interval and any clinical interventions between index test and reference standard	Section 2.1 Study design
Test results	23	Cross tabulation of the index test results (or their distribution) by the results of the reference standard	Section 3.2 Diagnostic performance of DWIBS and CT, Table 2, Table S3, Table S4
	24	Estimates of diagnostic accuracy and their precision (such as 95% confidence intervals)	Section 3.2 Diagnostic performance of DWIBS and CT, Table 2
	25	Any adverse events from performing the index test or the reference standard	Section 3.1 Characteristics of the patients
DISCUSSION			
	26	Study limitations, including sources of potential bias, statistical uncertainty, and generalisability	Discussion (Section 4)
	27	Implications for practice, including the intended use and clinical role of the	Discussion (Section 4)

		**index test**	
**OTHER INFORMATION**			
	**28**	**Registration number and name of registry**	**Section 2.1 Study design**
	**29**	**Where the full study protocol can be accessed**	**Section 2.1 Study design**
	**30**	**Sources of funding and other support; role of funders**	**Funding**

Table S2. Characteristics of cases enrolled due to suspected infectious aortitis

ID	Diagnosis	Periaortic CT findings	Clinical presentation	Microbiology, Microscopy	CRP, mg/dL	Leukocyte count, /µL	Procalcitonin, ng/mL	Reaction to antibiotics	Enhanced CT	Primary Analysis	Sensitivity Analysis
1	IAA (thoracic)	Fluid, dilation, saccular	Pain, fever	Negative	15.4	8400	0.06	y	y	Positive	Negative
2	IAA (thoracis/ paravisceral)	Fluid, saccular	Pain, fever	*Clostridium perfringens* (BC), Leukocyte (tissue)	12.7	8300	0.05	y	y	Positive	Positive
3	AGI (thoracic)	Fluid, saccular	Pain, fever	*Streptococcus anginous* (BC, tissue)	24.68	12100	0.81	y	y	Positive	Positive
4	Postoperative change	Fluid, saccular	None	Leukocyte (tissue)	9.88	8500	0.12	y	y	Negative	Positive
5	Closing aortic dissection	Dilation	Pain, fever	Negative	26.53	6100	1.57	N/A	y	Negative	Positive
+6	IAA (abdominal)	Fluid, dilation, saccular	Pain, fever	Leukocyte (tissue)	8.3	9200	0.06	y	y	Positive	Positive
7	IE	None	Pain, fever	*Streptococcus agalactiae* (BC)	23.44	31800	20.97	y	n	Negative	Negative
8	IAA (thoracic)	Fluid, dilation, saccular	Pain, fever	Negative	9.37	11600	0.04	y	y	Positive	Negative
9	Spondylitis	None	Pain, fever	*Enterococcus faecalis*	10.53	10400	0.08	y	n	Negative	Negative
10	IE	None	Fever, shock	*Staphylococcus aureus* (BC)	21.38	5200	5.04	y	y	Negative	Negative
11	AGI (thoracic)	Fluid	Fever, shock	*Pseudomonas aeruginosa* (BC)	25.98	12000	5.62	same day death	n	Positive	Negative
12	AA (paravisceral)	Saccular	None	Negative	2.46	6400	0.13	N/A	y	Negative	Negative
13	AGI (thoracic)	Fluid, dilation	Fever	Negative	20.24	11100	1.96	y	n	Positive	Negative
14	AGI (thoracic)	Fluid, dilation	Fever	*Staphylococcus aureus* (BC)	18.4	11980	0.59	y	y	Positive	Negative
15	Postoperative change	Fluid	None	Negative	0.04	0.3	0.04	N/A	n	Negative	Negative
16	IE	None	Pain, fever	Negative	14.18	12000	0.03	y	n	Negative	Negative

ID	Diagnosis	Periaortic CT findings	Clinical presentation	Microbiology, Microscopy	CRP, mg/dL	Leukocyte count, /µL	Procalcitonin, ng/mL	Reaction to antibiotics	Enhanced CT	Primary Analysis	Sensitivity Analysis
**17**	Postoperative wound infection	None	None	CNS (subcutaneous abscess)	6.45	10000	0.02	y	y	Negative	Negative
**18**	Postoperative change	None	Pain	*Corynebacterium spp.* (BC)	12.62	3100	2.51	n	n	Negative	Negative
**19**	Postoperative wound infection	None	Pain, fever	Negative	10.38	8300	0.03	y	y	Negative	Negative
**20**	AGI (Abdominal)	Fluid	Pain, fever	*Streptococcus anginous* (BC, tissue)	23.55	16200	4.71	n	n	Positive	Positive
**21**	Postoperative change	None	Fever	*Corynebacterium spp.* (BC)	23.07	7800	5.84	y	y	Negative	Negative
**22**	SMA dissection	None	Pain	Negative	7.81	5400	0.03	y	y	Negative	Negative
**23**	SMA dissection	None	Pain	Negative	0.06	4400	0.03	N/A	y	Negative	Negative
**24**	Giant cell arteritis	None	Pain, fever	Negative	18.8	9600	0.15	N/A	y	Negative	Negative
**25**	AA (thoracic)	None	None	*Cutibacterium acnes* (BC)	4.32	4500	0.4	N/A	n	Negative	Negative
**26**	IAA (thoracic)	Fluid, dilation, saccular	Fever	*Klebsiella pneumoniae* (BC)	17.31	13100	0.27	y	y	Positive	Negative
**27**	IAA (thoracic)	Fluid, dilation, saccular	Pain, fever	*Escherichia coli* (BC)	25.11	14400	0.41	y	y	Positive	Negative
**28**	IAA (abdominal)	Fluid, dilation, saccular	Pain, fever	*Streptococcus agalactiae* (BC)	13.15	18100	3.47	y	y	Positive	Negative
**29**	IAA (thoracic)	Fluid, saccular	Pain, fever	*Staphylococcus aureus* (BC)	26.32	13200	1.12	y	y	Positive	Negative
**30**	AGI (thoracic)	Fluid, gas	Fever	Negative	17.31	13100	0.27	y	n	Positive	Positive
**31**	AA (abdominal)	Saccular	None	Negative	7.45	4600	0.11	N/A	n	Negative	Negative
**32**	IAA (thoracic)	Fluid, saccular	Pain, fever	*Escherichia coli* (BC, tissue)	17.32	15000	0.51	Next day surgery	y	Positive	Positive

ID	Diagnosis	Periaortic CT findings	Clinical presentation	Microbiology, Microscopy	CRP, mg/dL	Leukocyte count, /µL	Procalcitonin, ng/mL	Reaction to antibiotics	Enhanced CT	Primary Analysis	Sensitivity Analysis

ID , patient identification; IAA, infectious aortic aneurysm; AGI, aortic graft infection; IE, infectious endocarditis; SMA, superior mesenteric artery; AA ,aortic aneurysm; CT, computed tomography; BC, blood culture; CNS, coagulase negative *Staphylococci*; *spp.*, species; CRP, C-reactive protein; y, yes; n, no; N/A, not applicable. Positive/Negative classifications refer to the reference standard diagnosis for each analysis (Infectious aortitis for primary analysis, modified for sensitivity analysis).

Table S3. Comparison of each evaluation method in diffusion-weighted whole-body imaging with background body signal suppression

	TP	FP	TN	FN	Sensitivity	Specificity	PPV	NPV	AUC
DWIBS (aorta)[a]	14	9	8	1	93.3 (68.1–99.8)	47.1 (23.0–72.2)	60.9 (38.5–80.3)	88.9 (51.8–99.7)	0.70 (0.56–0.84)
DWIBS (spinal cord)[b]	14	4	13	1	93.3 (68.1–99.8)	76.5 (50.1–93.2)	77.8 (52.4–93.6)	92.9 (66.1–99.8)	0.85 (0.73–0.97)
DWIBS (ADC Reader 1)[c]	11	7	0	1	91.7 (64.6–98.5)	0.0 (0.0–35.4)	61.1 (38.6–79.7)	0.0 (0.0-79.4)	0.77 (0.55–0.77)
DWIBS (ADC Reader 2)[c]	9	7	0	3	75.0 (46.8–91.1)	0.0 (0.0–35.4)	56.3 (33.2–76.9)	0.0 (0.0-56.2)	0.81 (0.60–0.81)

n, % (% confidence interval); Abbreviations: TP, true positive; FP, false positive; TN, true negative; PPV, positive predictive value; NPV, negative predictive value; AUC, area under the curve; ADC, apparent diffusion coefficient; DWIBS, diffusion-weighted whole-body imaging with background suppression; CT, computed tomography

[a]aorta was used as a reference, [b]spinal cord was used as a reference, [c]after calculating the AUC, a cutoff value of 0.7 was set and evaluated.

Table S4. Supplement to the character baseline: Reading findings by readers 1 and 2

Imaging modality	Reader	Overall<br>N = 32	Infectious Aortitis<br>N = 15	Non-Infectious Aortitis<br>N = 17
DWIBS (spinal cord)	Reader 1	16 (50.0)	13 (86.7)	3 (17.6)
	Reader 2	18 (56.3)	14 (93.3)	4 (23.5)
DWIBS (aorta)	Reader 1	22 (68.8)	14 (93.3)	8 (47.1)
	Reader 2	22 (68.8)	14 (93.3)	8 (47.1)
NCCT	Reader 1	19 (59.3)	14 (93.3)	5 (29.4)
	Reader 2	22 (68.8)	14 (93.3)	8 (47.1)

n (%); Abbreviations: DWIBS, diffusion-weighted whole-body imaging with background suppression; NCCT, non-contrast computed tomography

Table S5. Diagnostic performance of DWIBS and NCCT stratified by aortic pathology and postoperative phase.

	Modality	TP	FP	TN	FN	Sensitivity	Specificity	PPV	NPV	AUC
Native	DWIBS (spinal cord)	8	0	6	1	88.9% (56.5-98.0)	100% (61.0-100)	100% (67.6-100)	85.7% (48.7-97.4)	0.94 (0.82-1.00)
N = 15	NCCT	9	3	3	0	100% (70.1-100)	50.0% (18.8-81.2)	75.0% (46.8-91.1)	100% (43.9-100)	0.75 (0.50-1.00)
Artificial (all)	DWIBS (spinal cord)	6	4	7	0	100% (61.0-100)	63.6% (35.4-84.8)	60.0% (31.3-83.2)	100% (64.6-100)	0.82 (0.59-1.00)
N = 17	NCCT	5	2	9	1	83.3% (43.6-97.0)	81.8% (52.3-94.9)	71.4% (35.9-91.8)	90.0% (59.6-98.2)	0.83 (0.60-1.00)
Aritificail (<3months)	DWIBS (spinal cord)	0	3	2	0	N/A	40.0% (11.8-76.9)	0.0% (0.0-56.1)	100% (34.2-100)	N/A
N = 5	NCCT	0	0	5	0	N/A	100% (56.6-100)	N/A	100% (56.6-100)	N/A
Aritificail (>3months)	DWIBS (spinal cord)	6	1	5	0	100% (61.0-100)	83.3% (43.6-97.0)	85.7% (48.7-97.4)	100% (56.6-100)	0.92 (0.74-1.00)
N = 12	NCCT	5	2	4	1	83.3% (43.6-97.0)	66.7% (30.0-90.3)	71.4% (35.9-91.8)	80.0% (37.6-96.4)	0.75 (0.46-1.00)

n, % (% confidence interval); Abbreviations: TP, true positive; FP, false positive; TN, true negative; PPV, positive predictive value; NPV, negative predictive value; AUC, area under the curve; ADC, apparent diffusion coefficient; DWIBS, diffusion-weighted whole-body imaging with background suppression; CT, computed tomography

Figure S1. AUC curves for each reader when using ADC values

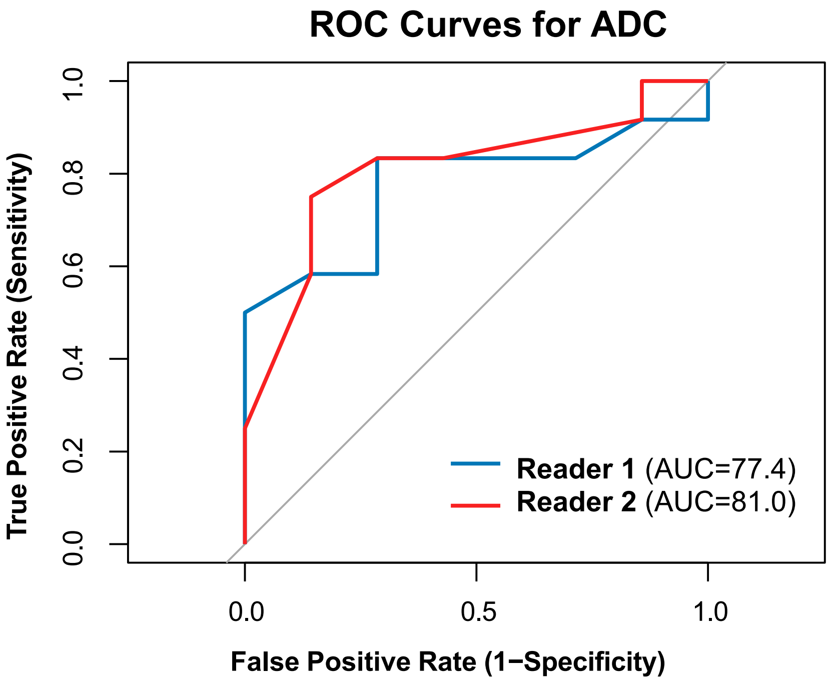

Figure S2. Comparison of diagnostic performance in subgroup

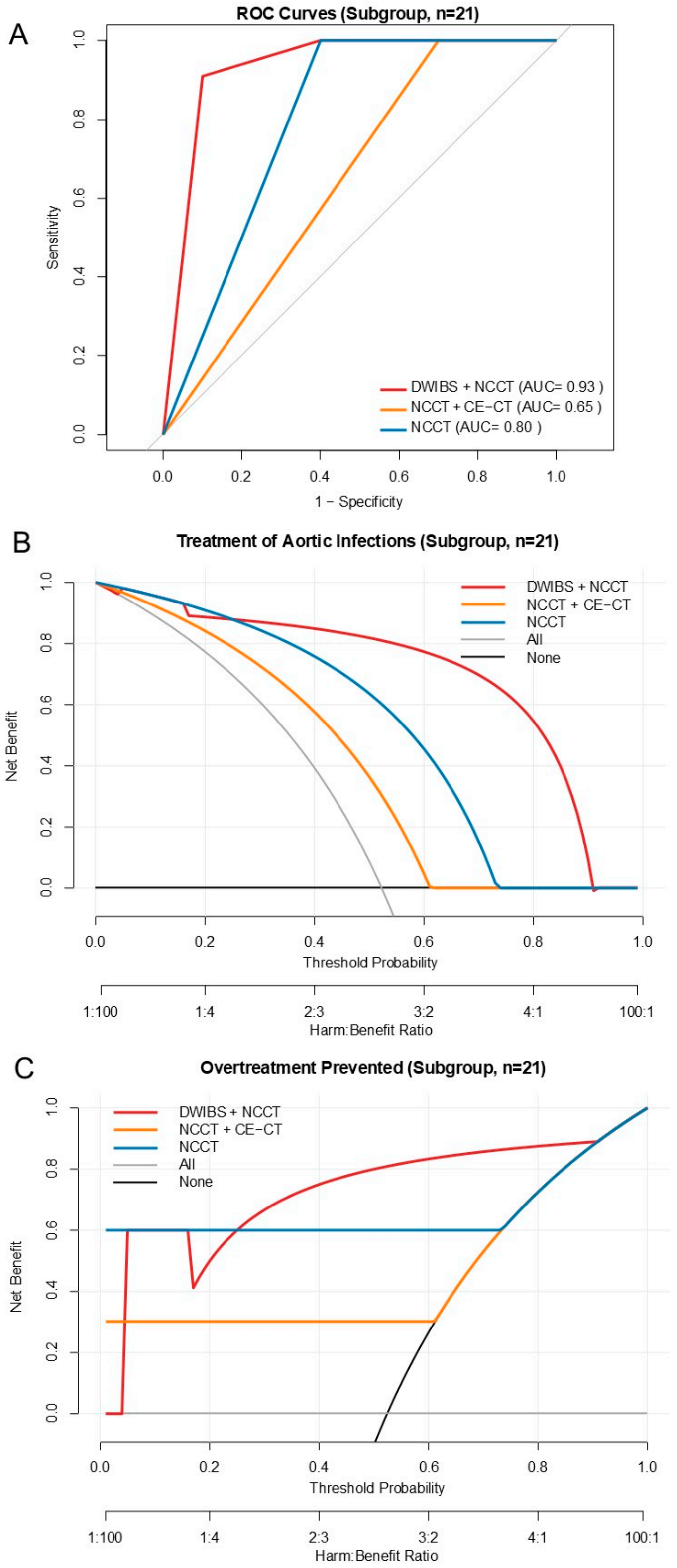

Supplement: Supplementary file 1 [file diagnostics-16-00225-s001.zip › diagnostics-4036612-supplementary.pdf]
